# Comparison of Inflammatory Cytokine Levels between Single-Port and Three-Port Thoracoscopic Lobectomy in the Treatment of Non-Small-Cell Lung Cancer

**DOI:** 10.1155/2022/3240252

**Published:** 2022-07-30

**Authors:** Song Liang, Xiao Yin, Yu Fu, Xinglin Li, Junchao Zhu, Ran Xu

**Affiliations:** ^1^Department of Thoracic Surgery, Shengjing Hospital, China Medical University, Shenyang, Liaoning, China; ^2^Department of Anesthesiology, Shengjing Hospital, China Medical University, Shenyang, Liaoning, China

## Abstract

**Introduction:**

Thoracoscopic minimally invasive surgery is the main method for the treatment of lung cancer. The reduction of surgical trauma can effectively reduce the intraoperative and postoperative inflammatory reaction. The aim of the study is to compare the intraoperative and postoperative inflammatory reactions in patients with non-small-cell lung cancer (NSCLC) treated by single-port thoracoscopic surgery and three-port thoracoscopic surgery.

**Methods:**

A total of 68 NSCLC patients (stages I and II) of thoracoscopic surgery were selected and randomly divided into two groups where they received either single-port thoracoscopic surgery or three-port thoracoscopic surgery. Intraoperative and postoperative serum inflammatory markers (C-reactive protein, CRP; serum amyloid A protein, SAA; and interleukin 6, IL-6) were detected using the enzyme-linked immunosorbent assay.

**Results:**

The CRP level of the single-port group was significantly lower than that of the three-port group during surgery, the first day after surgery, and third day after surgery (*P* < 0.05). The level of IL-6 in the single-port group was significantly lower than that in the three-port group during surgery on the first and third days after surgery (*P* < 0.05). The level of SAA in the single-port group was also significantly lower than that in the three-port group on the first and third days after surgery (*P* < 0.05).

**Conclusion:**

Compared with three-port thoracoscopic surgery, single-port thoracoscopic surgery could reduce the inflammatory response and improve the recovery of NSCLC patients. Single-port thoracoscopic surgery is worthy of further promotion in the current treatment field of NSCLC in terms of reducing intraoperative and postoperative inflammatory reactions.

## 1. Introduction

Many recent studies have shown that surgical trauma will result in an immunosuppressive state. Combined with the effect of surgical stress, it will often lead to metabolic changes, systemic inflammatory response, and other problems. The body resists and removes the harmful factors through the inflammatory reaction. However, an excessive reaction will damage the normal tissues and cells of the body. The smooth recovery of the body needs to balance the degree of inflammatory reaction [1–3]. Surgical patients will trigger different degrees of an inflammatory response due to different degrees of physical trauma, which runs through the process of postoperative recovery from the beginning of surgery and often prolongs the time of postoperative recovery. Reducing the intraoperative and postoperative inflammatory response of patients has always been the goal of surgeons, and a method is the reduction of surgical trauma.

The successful experience of the first single-port thoracoscopic wedge resection of the lung in 2004 provided us with a new surgical idea [4]. Subsequently, a large number of domestic and international studies and case reports show that single-port thoracoscopic surgery is safe and feasible in lobectomy and segmental resection. With the rapid development of single-port thoracoscopic surgery in recent years, the scope of application and clinical efficacy of the surgery is gradually becoming equivalent to the traditional three-port thoracoscopic surgery, which can ensure the safety of the operation and complete tumor resection, and has its own characteristics and advantages compared with the traditional three-port thoracoscopic surgery [5–9]. The reduction of incisions can significantly improve the postoperative pain and recovery of patients and wound healing [10–12].

In addition, single-port thoracoscopic surgery also has a subtle improvement in patients' intraoperative and postoperative inflammatory response compared with traditional three-port thoracoscopic surgery [13]. In this study, we compared and analyzed the intraoperative and postoperative inflammatory factor levels of single-port thoracoscopic surgery and three-port thoracoscopic surgery in patients with non-small-cell lung cancer (NSCLC). Through the comparison of the measured values, we further discussed the advantages of single-port thoracoscopic surgery in reducing inflammatory response and its application and promotion value in the treatment of patients with NSCLC compared with traditional three-port thoracoscopic surgery.

## 2. Materials and Methods

### 2.1. Clinical Cases

**Figure 1:**
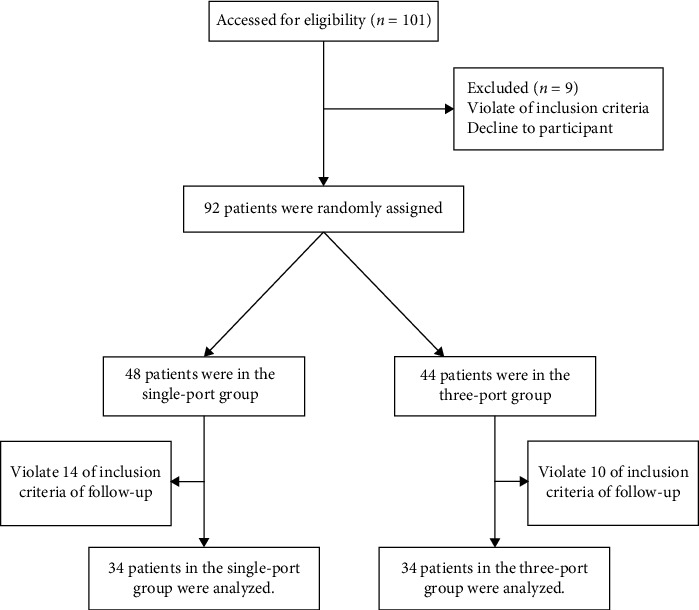
The flow chart on enrolled and excluded patients.

A total of 68 patients with NSCLC (stages 1 and 2) in our department from October 2021 to December 2021 were randomly divided into the single-port thoracoscopic experimental group (single-port group, 34 cases) and the three-port thoracoscopic control group (three-port group, 34 cases) (Figure 1). Inclusion criteria were as follows: (1) NSCLC was diagnosed by imaging examination (chest enhanced computed tomography or positron emission tomography-computed tomography) and biopsy pathology; (2) TNM stage was stages I and II; (3) the patient had indications of radical operation; (4) the patient had good cardiopulmonary, liver, kidney function, and no obvious surgical contraindication before operation; and (5) the preoperative inflammatory indexes of all patients were within the normal range. Exclusion criteria were as follows: (1) the thoracoscopic operation was converted to thoractomy, (2) the operation time was more than 3 hours, (3) blood vessel rupture occurred during the operation, and the bleeding amount was more than 200 mL, and (4) the patient had complications (the patient had fever exceeding 38.5°C, chest computed tomography confirmed intrapulmonary infection, incision infection, postoperative bleeding, requiring secondary thoracotomy, etc.). All patients adopted the same treatment and nursing process during the perioperative period. The study was registered on Clinicaltrials.gov (NCT05070026) before the patients were enrolled.

### 2.2. Therapeutic Method

Both groups were treated with double lumen endotracheal intubation and compound intravenous anesthesia, lying on the healthy side, one lung ventilated during the operation, and a cotton pad under the armpit. In the single-port group, a 4 ~ 5 cm incision was made between the fifth rib at the axillary front line. The protective sleeve was placed at the incision without the auxiliary operation port. The thoracoscopic rod and all surgical instruments entered the chest through this port, and a closed thoracic drainage tube was placed at the rear corner of the incision after operation. In the three-port group, two 1.5 cm incisions were made between the 7th rib of the axillary midline and the 6th and 7th rib of the posterior outer edge of the posterior axillary line, and small protective sleeves were placed, respectively, which were alternately used as observation ports and auxiliary operation ports. A 3 ~ 5 cm incision was made between the 3rd or 4th ribs of the axillary front as the main operation port, and the protective sleeve was placed on the incision. Standard lobectomy and mediastinal lymph node dissection were performed in both groups. At the end of the operation, the two groups of patients were given ropivacaine injection intercostal nerve blockade for pain relief, and the same peripheral intravenous pain relief pump was given for pain relief after operation, which was mainly composed of propacetamol hydrochloride for injection and nalbuphine hydrochloride injections.

As for the anesthesia procedure, analgesia and postoperative pain score were as follows:

Endotracheal intubation combined with intravenous anesthesia mainly includes the following steps:

1. Open the peripheral venous channel, do ECG monitoring, and prepare the anesthesia machine
2. When sufficient physiological needs are supplemented, sedatives, analgesics, and muscle relaxant are administered from peripheral intravenous channels
3. When the drug reaches the clinical peak concentration, perform tracheal intubation, auscultate both lungs, confirm that the tracheal tube is in the airway, and connect to the anesthesia machine

Both groups of patients used the same anesthetic drugs, and the dose was adjusted according to the patient's weight. The specific drugs included the followings: sufentanil, propofol injection, muscle relaxant rocuronide, remifentanil hydrochloride for injection, and sevoflurane.

The patients in both groups were given intercostal nerve blockade before chest closure. Currently, 0.375% ropivacaine was used to block the intercostal nerve between each incision and its upper and lower ribs, about 4 ml per intercostal space. Postoperative pain relief measures mainly include intravenous pain relief pumps, of which the main pain relief ingredient is nalbuphine hydrochloride injection, usually 20 mg per patient.

Collection of postoperative pain scores was as follows:

Each ward has a visual analog scale (VAS), which is explained to the patient by the doctor after surgery.

1. 0 point: no pain
2. Less than 3 points: slight pain, tolerable
3. 4 -6 points: the patient has pain which affects sleep but can still be tolerated
4. 7-10 points: the patient has severe pain and unbearable pain, affecting appetite and sleep

According to the above criteria, the doctor asks the patient about the pain level through a survey and asks the patient to draw a cross on the line that best reflects pain level.

As for the discharge criteria, postoperative patients will return to the ward with thoracic drainage buckets, and the thoracic drainage volume will be measured every day. When the swelling is good and there is little or no exudation, the thoracic drainage tube can be removed, followed by observation for 24 hours. Patients who have no adverse reactions such as fever, severe cough and expectoration, nausea and vomiting, and the results of the blood routine examination are satisfactory will be arranged for discharge.

### 2.3. Serum Specimen

Fasting elbow vein blood samples (3 ml) were taken at five time points (two days before operation, 30 minutes after beginning of operation, the end of operation, one day after operation, and three days after operation). The blood samples were centrifuged for 10 minutes at a rate of 3000 r/min. The upper serum was taken by pipette and stored in the serum anticoagulant centrifuge tube in a -80°C freezer.

### 2.4. Enzyme-Linked Immunosorbent Assay (ELISA)

The concentrations of C-reactive protein (CRP), serum amyloid A protein (SAA), and interleukin 6 (IL-6) in the serum specimens were detected using ELISA kits (SEA821Hu, SEA885Hu, and SEA079Hu) from Cloud-Clone Company. All operations were performed according to the manufacturers' protocol. After the experiment, any drops of water and fingerprints on the bottom of the plate were removed, and the absence of bubbles on the surface of the liquid was confirmed. Then, the microplate reader was run, and measurements were conducted immediately at 450 nm. A standard curve was drawn according to the O.D. value of the standard (*X*-axis) against the known concentration of the standard (*Y*-axis). The concentrations were calculated from the standard curve according to the sample O.D. value.

### 2.5. Statistical Analysis

The data in this study was analyzed using SPSS 24.0 (IBM Corp., Armonk, NY, USA) and GraphPad Prism 5 (GraphPad Software, San Diego, CA, USA). The measurement data was expressed in mean ± standard deviation. Any differences between groups were analyzed by *t*-test. A *P* value of <0.05 was considered to be statistically significant.

## 3. Results

### 3.1. Comparison of Clinical Basic Information between the Two Groups

There was no significant difference in age, gender, underlying disease, resection site, and postoperative pathological classification between the two groups (*P* > 0.05) (Table 1).

### 3.2. Comparison of Perioperative Indicators and Complications between the Two Groups of Patients

There was no significant difference in the operation time, intraoperative blood loss, and postoperative extubation time between the two groups (*P* > 0.05), but the two groups have significant difference in postoperative hospitalization time and the VAS pain score of the first postoperative day and the third postoperative day. The single-port group was better than that of the three-port group, and the difference was statistically significant (*P* < 0.05), as shown in Table 2. There was no significant difference in the incidence of postoperative complications between the single-port group and the three-port group, as shown in Table 3.

### 3.3. Comparison of CRP between the Two Groups at Different Time Points during Treatment

**Figure 2:**
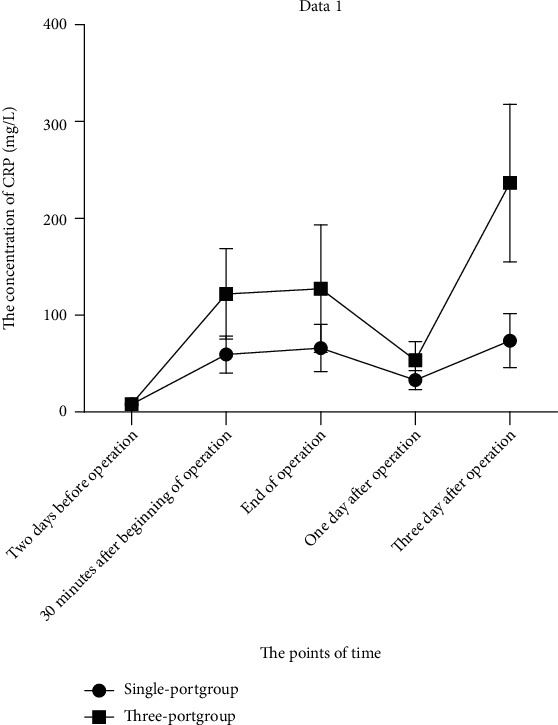
The comparison of CRP between two groups in different time points during treatment. ^∗^*P* < 0.05 vs. three-port group at the same point of time.

As shown in Figure 2 and Table 4, there was no significant difference in CRP levels between the two groups two days before the operation (*P* > 0.05). In addition, CRP levels increased significantly during surgery compared with presurgery and were higher in the three-port group than in the single-port group (*P* < 0.05). Similarly, CRP was higher in the three-port group than in the single-port group after surgery (*P* < 0.05).

### 3.4. Comparison of SAA between the Two Groups at Different Time Points during Treatment

**Figure 3:**
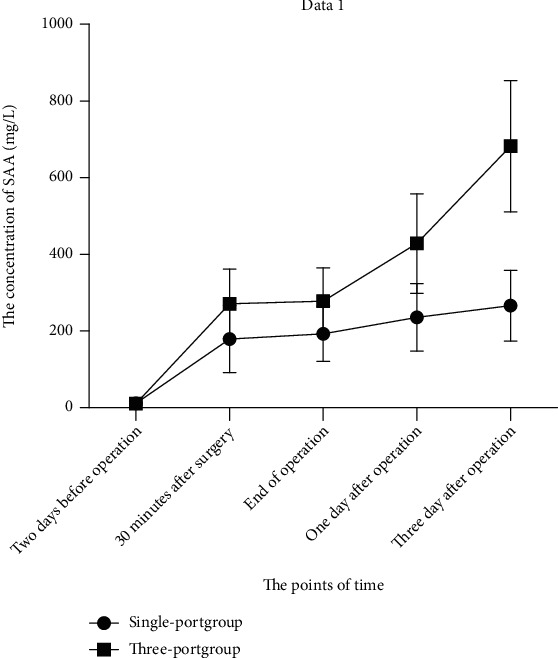
The comparison of SAA between two groups in different time points during treatment. ^∗^*P* < 0.05 vs. three-port group at the same point of time.

The preoperative SAA levels in the single-port group showed no significant difference when compared with the three-port group (Figure 3 and Table 5, *P* > 0.05). The intraoperative SAA levels during surgery rose in comparison to preoperative SAA levels. Moreover, SAA levels in the three-port group were higher than that in the single-port group during operation (*P* < 0.05). Postoperative SAA levels in the single-port group were lower than that in the three-port group (*P* < 0.05).

### 3.5. Comparison of IL-6 between the Two Groups at Different Time Points during Treatment

**Figure 4:**
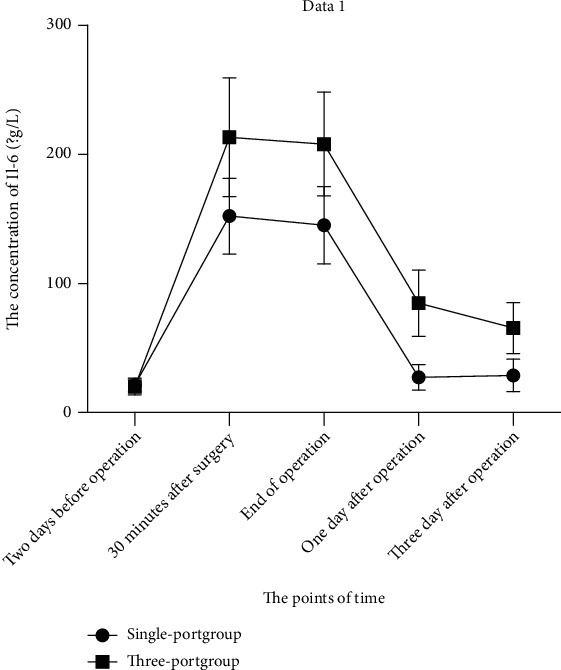
The comparison of IL-6 between two groups in different time points during treatment. ^∗^*P* < 0.05 vs. three-port group at the same point of time.

As shown in Figure 4 and Table 6, there was no significant difference in preoperative IL-6 levels between the single-port group and the three-port group (*P* > 0.05). In addition, IL-6 levels increased significantly during surgery compared with presurgery, and IL-6 levels were higher in the three-port group than in the single-port group (*P* < 0.05). Analogously, postoperative IL-6 levels in the single-port group were lower than that in the three-port group (*P* < 0.05).

### 3.6. Comparison of Postoperative Pain Score between the Two Groups at Different Time Points

**Figure 5:**
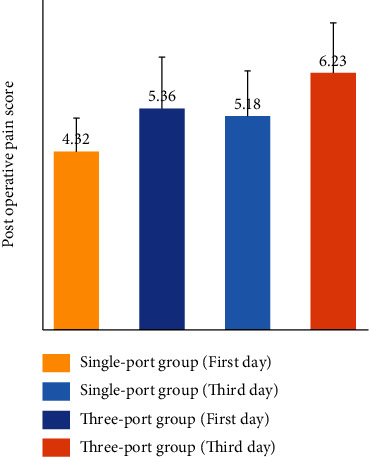
The comparison of postoperative pain score between two groups in different time points. ^∗^*P* < 0.05 vs. three-port group at the same point of time.

As shown in Figure 5 and Table 2,the postoperative pain score table showed that the pain score of the single-port group was lower than that of the three-port group on the first postoperative day and the third postoperative day, and the difference was statistically significant (*P* < 0.05).

## 4. Discussion

Lung cancer is a malignant tumor that threatens human health and life. Thoracoscopic surgery is one of the mainstream treatment methods at present [ 14–15]. Both traditional three-port thoracoscopic surgery and single-port thoracoscopic surgery can effectively remove tumors and surrounding lymph nodes and improve the prognosis of patients. However, the surgical trauma caused by the two surgeries is very different, which will affect the patients' level of inflammatory response reflected by the difference in the expression of inflammatory factors [16–18]. Single-port thoracoscopic surgery involves operating through a single port. On the premise of ensuring the operation quality, the shortening of the incision length and the reduction of the number of incisions can greatly reduce the injury to patients' nerves, muscles, and blood vessels [19–22]. In recent years, many studies have also shown that single-port thoracoscopic surgery can reduce the postoperative inflammatory response of patients compared with three-port thoracoscopic surgery [22–24]. In this experiment, there were differences in postoperative pain scores between the two groups, indicating that the pain intensity of the two surgical methods was different under the premise of the same effect of analgesics. It may had some impact on the patient's inflammatory response, but it boils down to the different levels of trauma caused by the two different procedures. In addition, the postoperative pain scores of the patients were maintained at mild to moderate levels, indicating that the intraoperative and postoperative pain relief methods were effective and reliable.

CRP, one of the most typical inflammatory factors, is a polypeptide prohormone composed of multiple amino acids, which is a reactive protein in acute liver cells. In healthy people, the level of CRP in blood is relatively low and will rise sharply in the occurrence of acute trauma. Its level can objectively reflect the degree of injury caused by the body's response to surgical stress. Recent studies have shown that CRP levels during or after surgery could reflect the stress response of patients undergoing lung cancer surgery [25–26]. This study also proved that the CRP level in the single-port group was significantly lower than that in the three-port group, and the postoperative recovery speed and quality of life were also significantly better than that in the three-port group. The experimental data proved that the CRP level in the single-port group was significantly lower than that in the three-port group, and the postoperative recovery speed and living quality were significantly better than that in the three-port group.

SAA is a highly heterogeneous protein in the apolipoprotein family. As an acute reactive protein produced after liver cell injury, SAA will increase 1000 times within 4 ~ 6 h after the body is injured or infected [27–30]. SAA can objectively reflect the degree of inflammatory response and is a sensitive indicator of inflammation dissipation, as well as an intuitive reflection of postoperative recovery of patients [31]. In this study, the SAA level of the single-port group was significantly lower than that of the three-port group during the operation. The degree of postoperative inflammation dissipated in the single-port group was faster than that in the three-port group, which indirectly reflected a better postoperative recovery in the single-port group than the three-port group.

IL-6 is mainly produced by macrophages, monocytes, fibroblasts, and lymphocytes, among others. The serum level of IL-6 is very low under normal and healthy conditions. But under certain conditions, such as in the early stages of inflammation, the concentration of IL-6 can be increased up to 10,000 times. As an inflammatory cytokine, IL-6 plays an important role in acute inflammation and is a good index to reflect surgical trauma. IL-6 can also stimulate the proliferation and differentiation and improve the function of cells involved in the immune response. At the same time, the expression level of the IL-6 protein is negatively correlated with the survival time of patients. Its abnormal secretion and the changes of related signal pathways are very important for tumor proliferation, metastasis, and prognosis [32–33].

The results of this study showed the changes in IL-6 concentration, which increased at each time point during and after surgery compared with presurgery in both groups, indicating that the stress response of the body is caused by surgical strike. However, the intraoperative and postoperative IL-6 level of the single-port group was significantly lower than that of the three-port group, indicating that the single-port group had less trauma to the body, less inflammatory response, and less immunosuppression after surgery, along with a shorter acute stress time of the body. As a result, the single-port thoracoscopic surgery was more conducive to the recovery of the body after surgery.

In addition, IL-6 acts as an acute mediator involved in B cell stimulation. The peak of IL-6 is usually reached at 2 hours after operation and then decreased rapidly in patients without complications. CRP is one of the representatives of acute phase proteins, which provides a reliable screening test for acute phase reactants. CRP levels peak at 24 to 72 hours after surgery and may continue to rise for about 2 weeks. Therefore, IL-6 and CRP can be used as objective biochemical markers to reflect surgical tissue trauma.

The quality of life of cancer patients has become an important standard to evaluate the effect of tumor treatment. Recently, a large number of studies have shown that a widely used and recognized general compound intravenous anesthesia combined with epidural anesthesia can more effectively reduce surgical stress stimulation, promote hemodynamic stability, and reduce the incidence of anesthesia-related complications, thereby reducing postoperative inflammatory response [34–35].However, due to the limitation of the current medical level in China, this anesthesia technique has not been carried out in thoracic surgery, but this is the goal of our future development and further research.

The subjects of this study were NSCLC patients undergoing minimally invasive lung surgery. They were usually discharged from the hospital on the fourth to fifth day after surgery. Therefore, it is impossible to continue to monitor the subsequent changes of inflammatory factors and the speed of recovery of patients and further compare the effects of the two surgical methods on patients.

In conclusion, compared with three-port thoracoscopic surgery, single-port thoracoscopic surgery has significant advantages in reducing intraoperative stress response, reducing postoperative inflammatory response, and accelerating the dissipation of inflammation. Single-port thoracoscopic surgery is worthy of further promotion in the current treatment field of NSCLC in terms of reducing intraoperative and postoperative inflammatory reactions.

## Figures and Tables

**Table 1:** The comparison of clinical basic information between the two groups.

Clinical basic information		Single-port group	Three-port group	*t*/*χ*^2^ value	*P* value
Age (year)		57.38 + 10.03	60.88 + 9.78	1.46	>0.05
Gender	Male	14	12	0.249	>0.05
	Female	20	22		
Hypertension	Yes	11	12	0.066	>0.05
	No	23	22		
Coronary heart disease	Yes	1	2	0.349	>0.05
	No	33	32		
Diabetes	Yes	4	6	0.469	>0.05
	No	30	28		
Tumor location	Right upper	12	15	0.775	>0.05
	Right middle	2	2		
	Right lower	5	4		
	Left upper	8	8		
	Left lower	7	5		
Histology	Adenocarcinoma	30	32	0.728	>0.05
	Squamous	2	1		
	Others	2			

**Table 2:** The comparison of perioperative indicators between the two groups.

Perioperative indicators	Single-port group	Three-port group	*t* value	*P* value
Operative time (min)	120.53 ± 35.87	108.67 ± 39.78	1.29	>0.05
Intraoperative blood loss (mL)	74.36 ± 25.43	72.31 ± 27.64	0.318	>0.05
Postoperative extubation time (d)	2.40 ± 0.80	2.67 ± 0.71	1.47	>0.05
Postoperative hospitalization (d)	4.73 ± 0.77	5.07 ± 0.68	1.93	<0.05
Postoperative pain score (first day)	4.32 ± 0.81	5.36 ± 1.25	4.07	<0.05
Postoperative pain score (third day)	5.18 ± 1.10	6.23 ± 1.21	3.74	<0.05

**Table 3:** The comparison of postoperative complications between the two groups.

	Pleural effusion	Subcutaneous emphysema	Pulmonary atelectasis	Atrial fibrillation	Infection of incisional wound	Lung leak	Total
Single-port group	1	1	1	4	0	0	7
Three-port group	1	2	1	4	1	0	9

**Table 4:** The comparison of CRP between two groups in different time points during treatment.

Point of time	Single-port group (mg/L)	Three-port group (mg/L)	*t* value	*P* value
Two days before operation	7.68 ± 1.36	8.01 ± 1.41	0.98	>0.05
30 minutes after surgery	59.23 ± 19.21	121.86 ± 46.70	7.23	<0.05
End of operation	65.97 ± 24.52	127.32 ± 65.93	5.09	<0.05
One days after operation	32.81 ± 9.90	53.23 ± 19.45	5.46	<0.05
Three days after operation	73.61 ± 28.02	236.42 ± 81.31	11.04	<0.05

**Table 5:** The comparison of SAA between two groups in different time points during treatment.

Point of time	Single-port group (mg/L)	Three-port group (mg/L)	*t* value	*P* value
Two days before operation	11.52 ± 6.25	10.83 ± 6.06	0.46	>0.05
30 minutes after surgery	179.23 ± 87.28	271.27 ± 90.25	4.28	<0.05
End of operation	192.74 ± 71.86	277.93 ± 86.49	4.42	<0.05
One days after operation	235.42 ± 88.01	428.26 ± 130.12	7.16	<0.05
Three days after operation	265.92 ± 92.51	682.05 ± 171.24	12.47	<0.05

**Table 6:** The comparison of IL-6 between two groups in different time points during treatment.

Point of time	Single-port group (*μ*g/L)	Three-port group (*μ*g/L)	*t* value	*P* value
Two days before operation	21.26 ± 5.25	19.86 ± 6.37	0.98	>0.05
30 minutes after surgery	152.15 ± 29.42	213.20 ± 46.07	6.51	<0.05
End of operation	145.06 ± 29.83	207.93 ± 40.18	7.33	<0.05
One days after operation	27.34 ± 9.87	84.67 ± 25.61	12.18	<0.05
Three days after operation	28.81 ± 12.50	65.52 ± 19.74	9.16	<0.05

## Data Availability

The raw data supporting the conclusions of this article will be made available by the authors, without undue reservation.
